# Minute Impurities Contribute Significantly to Olfactory Receptor Ligand Studies: Tales from Testing the Vibration Theory

**DOI:** 10.1523/ENEURO.0070-17.2017

**Published:** 2017-06-19

**Authors:** M. Paoli, D. Münch, A. Haase, E. Skoulakis, L. Turin, C. G. Galizia

**Affiliations:** 1 Neurobiology, University of Konstanz, Konstanz, 78457, Germany; 2 Department of Physics and Center for Mind/Brain Sciences, University of Trento, Povo, TN 38123, Italy; 3 Division of Neuroscience, Biomedical Sciences Research Centre Alexander Fleming, Vari 16672, Greece

**Keywords:** deuteration, olfactory receptors, vibration theory

## Abstract

Several studies have attempted to test the vibrational hypothesis of odorant receptor activation in behavioral and physiological studies using deuterated compounds as odorants. The results have been mixed. Here, we attempted to test how deuterated compounds activate odorant receptors using calcium imaging of the fruit fly antennal lobe. We found specific activation of one area of the antennal lobe corresponding to inputs from a specific receptor. However, upon more detailed analysis, we discovered that an impurity of 0.0006% ethyl acetate in a chemical sample of benzaldehyde-d_5_ was entirely responsible for a sizable odorant-evoked response in *Drosophila melanogaster* olfactory receptor cells expressing dOr42b. Without gas chromatographic purification within the experimental setup, this impurity would have created a difference in the responses of deuterated and nondeuterated benzaldehyde, suggesting that dOr42b be a vibration sensitive receptor, which we show here not to be the case. Our results point to a broad problem in the literature on use of non-GC-pure compounds to test receptor selectivity, and we suggest how the limitations can be overcome in future studies.

## Visual Abstract

**Figure:**
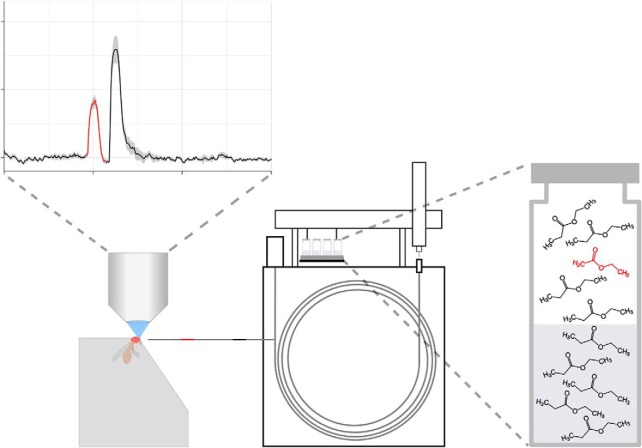

## Significance Statement

How exactly odorant receptors create selectivity for some odorants against the vast number of alternatives remains as yet unclear and is generally probed by measuring responses to different substances. Chemical senses are highly sensitive to minute amounts of odorants in the environment. Therefore, when testing the responses of olfactory receptors, substances of highest purity are used, generally 95% or 99%, i.e., with impurities of 5% or 1%. We report a case where an impurity of 0.0006% was sufficient to explain the full response of an olfactory receptor in a test situation. We demonstrate why all experiments investigating the selectivity of odor receptors have to be performed with gas-chromatography-purified odors to eliminated potential impurity artifacts.

## Introduction

How odorants interact with receptors remains elusive: a key-lock system has been proposed early on (Amoore, 1963), but this does not yet explain how a transduction cascade is activated (i.e., how the fitting key is turned inside the lock). Different mechanisms have been proposed, including the involvement of metal ions creating metalloproteins (Turin, 1996; Wang et al., 2003; Duan et al., 2012), and electron tunneling in resonance with molecular vibrations (Turin, 1996).

Crystallography is the most direct approach to studying receptor-ligand interaction, but only few examples exist, including the cholinergic receptor (Warne et al., 2008) and photoreceptors (Palczewski et al., 2000; Standfuss et al., 2011). No olfactory receptor has been analyzed in this way yet. An alternative approach relies on modeling the binding pocket (Guo and Kim, 2010). Here, large sets of odor-response data are necessary, ideally recorded in a hypothesis-free approach. However, in both cases, the result consists in an estimate for the shape of the binding pocket, but not yet in a mechanism of how the receptor is activated. Dedicated, hypothesis-driven studies are better suited to this end: if vibrations are to be tested, the task would be to find a receptor that does respond to one vibration frequency, and not to another.

Deuterated substances offer an ideal possibility to test whether molecular vibrations contribute to activating olfactory receptors. When hydrogen (H) is replaced by deuterium (D) in a molecule, the chemical properties do not change, but a new vibration range is added. For example, the C-D bond has a vibration at around 2150 cm^−1^, which is not present in a molecule lacking deuterium. Deuterium can also add other vibrations: the ring in benzaldehyde-d_5_ creates a collective out-of-plane vibration around 550 cm^−1^ (Klika, 2013; Paoli et al., 2016). The logic of these experiments is that, if an animal can differentiate between a deuterated and a nondeuterated substance that otherwise are equal (say, between benzaldehyde and its deuterated form, which smell almond like to humans), vibrations must play a role, since that is the main physical factor that differentiates the two odorant stimuli. This hypothesis has been tested in a variety of studies, using humans, fruit-flies, honeybees and other animals, and using paradigms including behavior and physiology (Haffenden et al., 2001; Keller and Vosshall, 2004; Franco et al., 2011; Bittner et al., 2012; Gane et al., 2013; Gronenberg et al., 2014; Paoli et al., 2016). However, the results are contradictory, since some studies argue for and others against vibrations, leading to controversial discussions (Solov'yov et al., 2012; Block et al., 2015).

Another aspect to be considered is that olfactory receptor gene families are highly divergent. Even within single species, there are several unrelated families of olfactory receptors: in mammals, at least six different families have been reported (Fleischer et al., 2009; Greer et al., 2016), in insects, ionotropic receptors (IRs) and olfactory receptors (ORs) are two distinct families (Silbering et al., 2011). A hypothesis would be that a single family, or even a particular receptor, could use one or more activation mechanisms, e.g., vibration detection, size, etc., while others could respond to different odorant properties. Therefore, studying how responses to deuterated substances differ from nondeuterated substances is best done on single receptor types, rather than the whole olfactory system.

Receptors have broad or narrow response profiles (Galizia et al., 2010; Münch and Galizia, 2016), but even the latter respond to minor ligands when presented at a sufficiently high concentration. Optimal concentrations for eliciting responses in receptors can span many orders of magnitude. For example, Or22a in *Drosophila* has an EC_50_ of 10^−6.9^ for methyl hexanoate, and an EC_50_ of 10^−4.2^ for isoamyl acetate, and both dilutions create concentrations that *Drosophila* is easily exposed to in a natural environment (Pelz et al., 2006). The difference of several orders of magnitude between these two stimuli means that small amounts of impurities can have a strong effect on odor responses. Examples of single sensillum recordings where the responses were entirely due to impurities in commercial odorant sources have been published for moths (Stranden et al., 2003).

In this study, we combined these thoughts in an attempt to test the vibration theory of olfaction. First, we searched for a single receptor type that would show differential responses between deuterated and nondeuterated substances, and found one with an apparent difference. Results such as these have been published as evidence in favor of the vibrational theory. Next, we recorded the odorants' responses via a gas chromatograph, and found that in our case the difference was due to a minute contaminant (0.0006%, or 6 ppm). Finally, we show that adding the contaminant to the nondeuterated substances elicits a response similar to the one seen for the deuterated substance. We conclude that the results do not support the vibrational theory. Importantly, however, they do not disprove it either, rather, they show how important it is not only to use substances of highest purity, but indeed to purify substances on the spot using gas chromatography. As a corollary, the validity of data in studies on receptor-ligand interaction in general that have not used appropriate purification techniques needs to be reconsidered.

## Materials and Methods

### Animals

All recordings were performed on female *Drosophila melanogaster* fruit flies expressing either the calcium reporter G-CaMP5 (Akerboom et al., 2012) under the control of the olfactory co-receptor Orco (Orco-Gal4 > UAS-GCaMP5), or expressing the reporter GCaMP6m (Chen et al., 2013) in Or42b olfactory receptor neurons (Or42b-Gal4 > UAS-GCaMP6m). Calcium reporter driver lines were obtained from the Bloomington Stockcenter (RRID:BDSC_42038 and RRID:BDSC_42748), Or42b-Gal4 (likely RRID:BDSC_9972), and Orco-Gal4 (likely RRID:BDSC_26818) flies were kindly provided by Veith Grabe and Silke Sachse (MPI for Chemical Ecology, Jena, Germany). Flies were kept at 25°C in a 12/12 h light/dark cycle at 60-70% RH. Animals were reared on standard medium (100 ml contain: 2.2 g of yeast, 11.8 g of sugar beet syrup, 0.9 g of agar, 5.5 g of cornmeal, 1 g of coarse cornmeal, and 0.5 ml of propionic acid).

### Animal preparation

For antennal lobe recordings flies were anesthetized on ice and placed into a custom-made holder. The head was fixed to the holder with low-melting wax, the antennae were gently pulled forward with a thin copper wire, and a polyethylene foil was placed on the head and sealed with bicomponent silicon (Kwik-Sil, WPI). A small window was cut through the foil and head cuticle, and the exposed brain was covered in saline solution (130 mM NaCl, 5 mM KCl, 2 mM MgCl_2_, 2 mM CaCl_2_, 36 mM sucrose, and 5 mM HEPES, pH 7.3; all chemicals from Sigma-Aldrich). Glands and tracheae were removed to allow optical access to the antennal lobe. For antenna recordings flies were mounted in custom-made holders. The head was fixed to the holder with a drop of low-melting wax. A half electron-microscopy grid was placed on top of the head, stabilizing the antenna by touching the second, but not the third, antennal segment. For details on the antennal lobe preparation, see (Silbering and Galizia, 2007; Silbering et al., 2008). For details on the antennal preparation, see Münch and Galizia (2016).

### Odorant preparation

Benzaldehyde-2,3,4,5,6-d_5_ was purchased at CDN isotopes (CAS: 14132-51-5, lot I240P14, isotopic enrichment 99%). All other odorants were purchased at Sigma-Aldrich in the highest purity available. Odorants used were: benzaldehyde (CAS: 100-52-7, lot STBD7798V, ≥99.5%), E2-hexenal (CAS: 6728-26-3, lot S28442V, 98%), ethyl acetate (CAS: 141-78-6, lot BCBR9070V, ≥99.9%), ethyl propionate (CAS: 105-37-3, lot BCBL5952V, ≥99.7%), ethyl butyrate (CAS: 105-54-4, lot BCBR7796V, ≥99.5%), propyl acetate (CAS: 109-60-4, lot BCBL5998V, ≥99.7%), ethyl (S)-(+)-3-hydroxybutyrate (CAS: 56816-01-4, lot BCBM4473V, 99%), 3-hexanone (CAS: 589-38-8, lot BCBJ8237V, 98%), beta-butyrolactone (CAS: 3068-88-0, lot MKBJ3709V, 98%), (±)-2-Hexanol (CAS: 626-93-7, lot MKBJ5626V, ≥ 98%), methyl acetate (CAS: 79-20-9, lot BCBN9450V, ≥99.9%), and 3-penten-2-one (CAS: 625-33-2, lot SHBC5346V, ≥70%). Pure substances were diluted in mineral oil (Sigma-Aldrich) at the indicated dilutions, and covered with Argon (Sauerstoffwerk Friedrichshafen GmbH) to avoid oxidation. Dilutions were prepared in 5 ml mineral oil (CAS: 8042-47-5; Acros Organics) in 20 ml head space vials covered with pure nitrogen to avoid oxidation (Sauerstoffwerk Friedrichshafen GmbH) and immediately sealed with a Teflon septum (Axel Semrau).

### Odorant delivery

A GC-FID system (TRACE GC Ultra, Thermo Fisher Scientific) in conjunction with an autosampler (PAL, CTC Switzerland) was used for odorant delivery. The autosampler was used to either inject headspace samples into the GC, or directly to the antenna, bypassing the GC system. For GC-coupled antenna measurements, 1 ml of headspace was injected into the GC at split mode with the injector temperature set to 200°C, the split flow to 15 ml/min and the split ratio to 10. The GC was equipped with an Optima 5 MS 30 m × 0.25 mm × 0.25 µm column (Macherey-Nagel). The flow of the carrier gas helium was set to 1.5 ml/min. The oven was held at 60°C for 1 min, then the temperature was increased to 200°C at 20°C/min, the final temperature was again held for 1 min. One half of the eluate was directed to the FID detector (set to 200°C) and the other half to the animal’s antenna via an olfactory detection port (either ODP3, Gerstel or Semrau). GC-FID trace and antennal trace alignment was calibrated using the response peak to ethyl acetate. FID data were recorded using Xcalibur software (Thermo Fisher Scientific). After each injection the syringe was washed with n-pentane (Merk KgaA), heated and flushed with clean air. For direct stimulations (bypassing the GC) a head space of 2 ml was injected in two 1 ml portions at time points 6 and 8.5 s with an injection speed of 1 ml/s into a continuous flow (60 ml/min) of purified air (two 1-s stimuli with 1.5-s gap). Stimuli arrived at the antenna with ∼750-ms delay due to delays in the autosampler and the flow. Therefore, stimulus onset was determined as 6.75 and 9.25 s. In the figures, *t* = 0 was set to correspond to the first stimulus onset. The stimulus was directed at the antenna of the animal via a Teflon tube (inner diameter, 2 mm; length, 39.5 cm, with the exit positioned ∼2 mm from the antenna). Between successive stimuli, the syringe was flushed with clean air. The intertrial interval was ∼2 min. For each animal, before odor delivery, responses to clean air and to mineral oil only were tested as controls.

### Calcium imaging

Calcium imaging of antenna (dendrites and somata of olfactory sensory neurons) and antennal lobes (axon terminals of olfactory sensory neurons) was performed on a setup consisting of a fluorescence microscope (BX51WI, Olympus) equipped with a 20× water immersion objective for antennal lobe recordings (Olympus XLUM Plan FI 20×/0.95) or with a 50× air lens without coverslip correction for antenna recordings (Olympus LM Plan FI 50×/0.5). Images were recorded with a CCD camera (SensiCam, PCO) with 4 × 4 pixel on-chip binning, which resulted in 160 × 120 pixel sized images for antennal lobe (AL) recordings or with 8 × 8 pixel on-chip binning, which resulted in 80 × 60 pixel sized images for antenna recordings. For AL measurements we recorded each stimulus for 20 s at a rate of 4 Hz using TILLvisION (TILL Photonics), GC-coupled antenna imaging was performed at 1 Hz for 9 min. A monochromator (Polychrome V, TILL Photonics) produced excitation light at a wavelength of 470 nm which was directed onto the antenna via a 500 nm low-pass filter and a 495 nm dichroic mirror. Emission light was filtered through a 505 nm high-pass emission filter.

**Figure 1.**
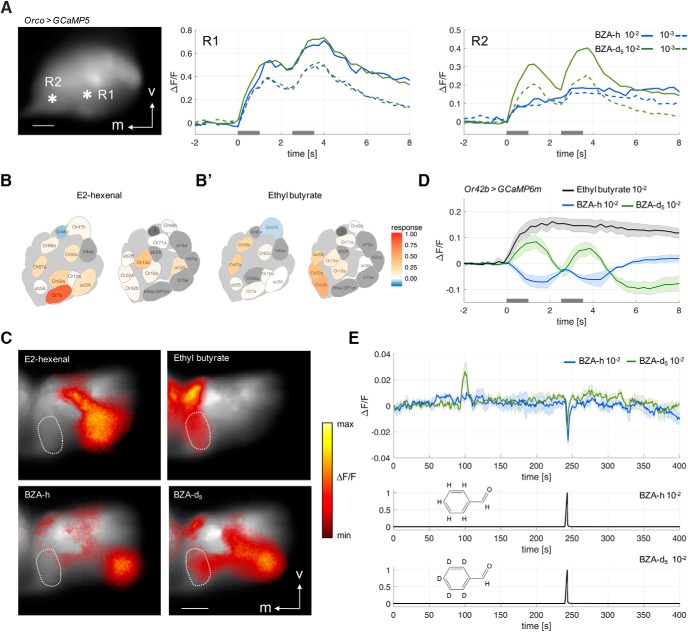
Apparent differential responses to deuterated and non-deuterated benzaldehyde. ***A***, Example of responses to benzaldehyde-d_5_ (green traces) and benzaldehyde-h (blue traces) at two different dilutions (dashed: 10^−3^, continuous 10^−2^) in two areas of the antennal lobe (R1 and R2). The left photograph indicates the position of R1 and R2 in the antennal lobe stained with the calcium sensor GCaMP5, the middle graph depicts the response time-traces in area R1, the right graph R2. Gray bars indicate stimulation times. Scale bar, 20 µm. ***B***, ***B'***, Spatial activity maps of the *Drosophila* antennal lobe for the odorants E2-hexenal and ethyl butyrate, taken from the DoOR database, http://neuro.uni.kn/door. ***C***, Spatial response patterns in the antennal lobe (false color) superimposed onto the morphologic view of the brain (grayscale). Responses to E2-hexenal, ethyl butyrate, and the two benzaldehydes (BZA-h and BZA-d_5_). Glomerulus DM1 innervated by dOr42b is circled with a dotted line. The midline of the brain is to the left, and the orientation of the brain corresponds to ***B***. Scale bar, 20 µm. ***D***, Calcium recording from neurons expressing Or42b in the DM1 glomerulus of the antennal lobe using the calcium sensor GCaMP6m. Stimuli were diluted to 10^−2^. Ethyl butyrate elicited long-lasting responses, which did not resolve the temporal structure of the double stimulus. Benzaldehyde-d_5_ elicited clear excitatory responses (calcium increases), while benzaldehyde-h elicited clear inhibitory responses (calcium concentration decreases) to each of the two odor pulses. Mean ± SEM (*N* = 3 animals). ***E***, Coupled GC-antennal lobe recordings in Or42b > GCaMP6m flies. The two bottom traces show the FID signal for the two benzaldehydes used, the top panel shows the mean response ± SEM to benzaldehyde-h (concentration 10^−2^, blue trace) and benzaldehyde-d_5_ (concentration 10^−2^, green trace, *N* = 3 animals). Both benzaldehydes show a clear calcium decrease in glomerulus DM1 at the elution time of benzaldehyde (approximately 240 s), but only benzaldehyde-d_5_ shows a strong calcium increase at elution time around 100 s.

**Figure 2.**
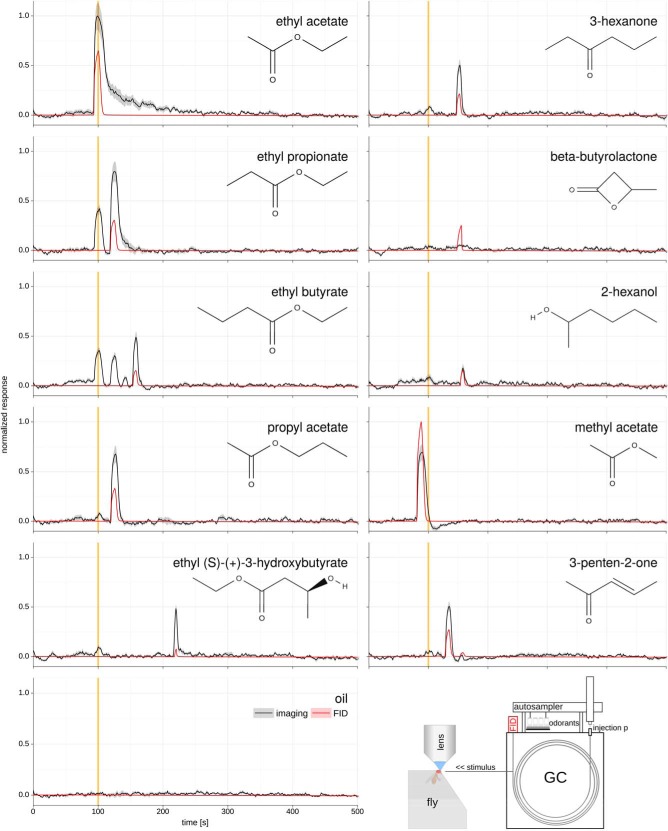
GC-Imaging recordings reveal minute impurities in commercial odorant sources. Each panel shows a GC-FID recording (red trace) and a simultaneous antenna calcium imaging trace from Or42b > GCaMP6m flies (black trace). All odors were injected as headspace samples at 10^−2^ dilution. The yellow bar indicates the elution time for ethyl acetate (100 s). A response in Or42b at that elution time is present in several samples (left column), but other impurities were also found (see response to ethyl butyrate). All traces: *N* = 4-5, average ± SEM For GC-FID traces, the error is smaller than the line width. Bottom right: schematic of the experimental setup.

**Figure 3.**
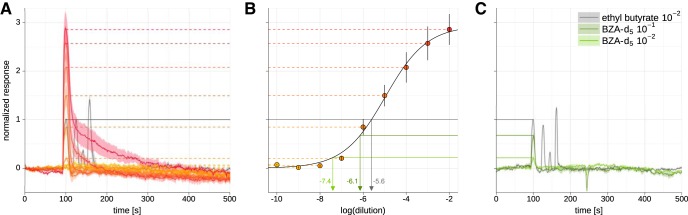
The impurity in benzaldehyde-d_5_ is 0.0006% ethyl acetate. ***A***, Responses to different concentrations of ethyl acetate in GC-Imaging of Or42b > GCaMP6m antennae (dilutions 10^−10^ to 10^−2^). Increasing concentrations are given in colors from orange-yellow to red. With increasing concentration, the response increases in size, but remains at the same elution time of approximately 100 s. At the highest concentrations, responses tail to the right. In gray, the response to 10^−2^ ethyl butyrate, which gives four response peaks, the first peak likely due to presence of ethyl acetate. All responses are normalized to the first response peak in ethyl butyrate. ***B***, Dose-response curve to ethyl acetate in GC-Imaging recordings. Peak responses are taken from ***A*** (dotted lines from the left). Responses have been fitted with a sigmoidal dose-response curve, EC_50_ is reached at a dilution of 10^−5.0^. Green lines from panel ***C*** indicate the response intensities found there, gray line the value of the first peak in the ethyl butyrate response. ***C***, GC-Imaging responses to our samples of benzaldehyde-d_5_ at a dilution of 10^−2^ (bright green) and 10^−1^ (dark green). At the elution time of benzaldehyde (approximately 240 s) both samples elicit a prominent concentration-dependent calcium decrease. At the elution time of ethyl acetate (approximately 100 s) both samples elicit a strong, concentration-dependent calcium increase. Traces have been normalized to the response to ethyl butyrate (gray trace). The concentration of the impurity can be extracted from the dose-response curve in ***B*** (green lines) as 10^−7.4^ and 10^−6.1^, for 10^−2^ and 10^−1^ dilution, respectively (*N* = 3 animals).

**Figure 4.**
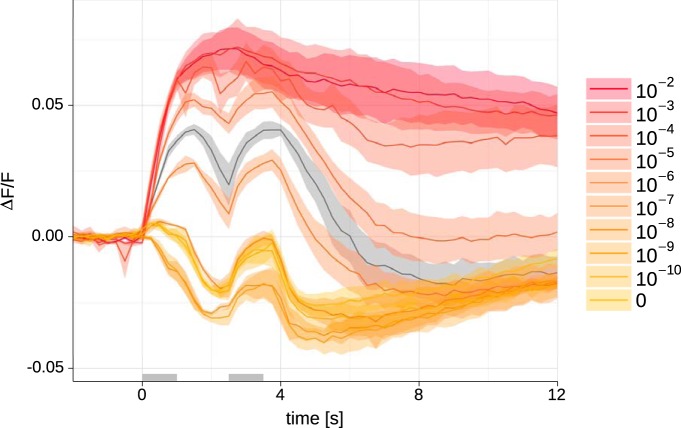
A minute impurity of ethyl acetate is sufficient to elicit a positive response to its mixture with benzaldehyde. We recorded antennal calcium responses in Or42b > GCaMP6m flies. Responses to benzaldehyde-h 10^−2^ were inhibitory. Gradually adding ethyl acetate in concentrations from 10^−10^ to 10^−2^ led to increasingly excitatory responses, in a dose-dependent manner (color-scale, see inset; for example, 0 in the legend means benzaldehyde-h at a dilution of 10^−2^; 10^−7^ in the legend means that ethyl acetate at a dilution of 10^−7^ was added to benzaldehyde-h at dilution 10^−2^, i.e., the relative concentration was 10^−5^). Gray: response to ethyl butyrate 10^−2^, for calibration. Odors were premixed in mineral oil to mimic the contamination situation, and delivered with a PAL multisampler. All traces: *N* = 3, average ± SEM. Gray bars indicate stimulation times.

Benzaldehyde-h/d_5_ antennal lobe measurements were performed in a total of *N* = 6 animals expressing Orco > GCaMP5 (Fig. 1*A*,*C*), and *N* = 3 animals expressing Or42b > GCaMP6m (Fig. 1*D*). The GC-coupled antenna recordings in Figure 2 are based on data from *N* = 5 animals expressing Or42b > GCaMP6m, dose-response data in Figure 3*A*,*B* are based on data from *N* = 5 animals expressing Or42b > GCaMP6m. GC-coupled antenna recordings of benzaldehyde-h/d_5_ were performed in a total of *N* = 3 animals expressing Or42b > GCaMP6m (Figs. 1*E*, 3*C*). Responses to blended benzaldehyde-h with increasing concentrations of “contaminant” were measured in *N* = 3 animals expressing Or42b > GCaMP6m (Fig. 4).

### Data analysis

Custom made R and Python scripts were used for data analysis. The Python-based ILTIS software (Georg Raiser, unpublished observations; https://github.com/grg2rsr/ILTIS) was used for calcium imaging visualization, baseline subtraction and normalization. Relative fluorescence change was calculated as *ΔF/F* = (*F_i_* - *F_0_*)/*F_0_* with *F_i_* being the fluorescence at frame *i* and *F_0_* being the mean fluorescence before stimulus onset. GC-antenna recordings were corrected for dye bleaching by fitting an exponential decay function of the form A * e^−x/B^ + C to each response trace, leaving out the parts of the trace where activity was recorded. Responses were calibrated across animals to the first response peak of ethyl butyrate, most likely 10^−5.6^ ethyl acetate (Fig. 3).

Dose-response curve (Fig. 3*B*) was obtained by least-squares fitting responses *R* at concentrations *c* with a sigmoidal logistic function of the form $R=R_{max}*\frac{1}{1+e^{-h*(c-EC50)}}$, with *R_max_* corresponding to maximum response asymptote, EC_50_ the half-effective dilution, and *h* the steepness (reminiscent of the Hill coefficient).

## Results

We used calcium imaging of the antennal lobe in the fruit fly *D. melanogaster* to record odorant evoked activity patterns. Specifically, we were interested in differences between the responses to benzaldehyde-h (normal benzaldehyde), and benzaldehyde-d_5_, where the hydrogen atoms of the benzene ring were replaced by deuterium. We expressed the calcium sensor GCaMP5 (Akerboom et al., 2012) under the control of the olfactory coreceptor Orco (GAL4-Orco > UAS-GCaMP5), and stimulated with two 1 s stimuli with a 1.5 s gap in between. Both normal and deuterated benzaldehyde elicited similar responses throughout the antennal lobe, with the strongest response in the dorsolateral area (Fig. 1*A*, area R1).

However, we also noted a dorsomedial area with clearly different responses to the two isotopomers, with apparent odorant elicited responses to benzaldehyde-d_5_, and no apparent responses to benzaldehyde-h (Fig. 1*A*, area R2). Therefore, we focused on this area because it could provide an important, clear test of the vibrational hypothesis. Using the antennal lobe atlas for *Drosophila* (Grabe et al., 2015), we identified two potential candidates for this area: glomerulus DM1, innervated by Or42b, and glomerulus DL5, innervated by Or7A. To confirm the identity of the putative isotope-sensitive area, we screened the DoOR database (Münch and Galizia, 2016) for two odorants that induced a strong response in either the DL5 or the DM1 glomerulus. For this purpose, we selected E2-hexenal (Fig. 1*B*) and ethyl butyrate (Fig. 1*B'* ). E2-hexenal gave a strong response in the dorsolateral area, corresponding to glomerulus DL5, which is innervated by axons from ORs expressing Or7A (Fig. 1*C*). Ethyl butyrate elicited responses more medially, corresponding to the area innervated by Or42b and Or22a (Fig. 1*C*). A comparison between the response patterns induced by the four odorants indicated a clear overlap between the dorsomedial area of the ethyl butyrate-induced signal, corresponding to glomerulus DM1, and the benzaldehyde-d_5_ responsive region (Fig. 1*C*, dotted line). Thus, we confirmed this area to be glomerulus DM1, innervated by Or42b. We then expressed the calcium sensor GCaMP6m (Chen et al., 2013) specifically in the Or42b receptor neurons (Or42b-GAL4 > UAS-GCaMP6m), and confirmed that Or42b responded to ethyl butyrate as well as to benzaldehyde-d_5_ (Fig. 1*D*). Responses to benzaldehyde-h, however, were inhibitory (Fig. 1*D*, blue trace).

To show more conclusively that the response of this glomerulus was due to benzaldehyde-d_5_, and to exclude that minor impurities could cause this difference between the two isotopomers, we coupled the imaging setup to a gas chromatograph outlet. With this experimental setup, response to either benzaldehyde-h or benzaldehyde-d_5_ was inhibitory at the elution time of benzaldehyde. However, we found a strong excitatory response to benzaldehyde-d_5_ at an earlier elution time, which was not present in the benzaldehyde-h recording (Fig. 1*E*). These results indicated that the apparent response to benzaldehyde-d_5_ in Or42b was due to some contaminating trace molecules. These data also suggested that the inhibitory response to benzaldehyde-d_5_ (as seen in Figure 1*D*) was masked by the contaminating substance.

Next, we sought to identify the impurity. Using the DoOR database (Münch and Galizia, 2016), we selected a set of best ligands for Or42b, purchased them at highest available purity, and measured their chemical purity using GC-FID (Fig. 2, red traces). With the exception of 3-penten-2-one, where we saw two peaks, all other substances only had a single detectable peak in the FID trace, with all minor peaks in the noise range. Next, we recorded the calcium responses in Or42b to the GC eluates. We found a strong response to ethyl acetate that decayed progressively after the stimulus, indicating receptor saturation. Similarly, ethyl propionate, propyl acetate, and ethyl (S)-(+)-3-hydroxybutyrate all elicited responses that decayed slowly after the stimulus had terminated, indicating some degree of saturation. Most importantly, however, we noted that ethyl propionate, ethyl butyrate, propyl acetate, and ethyl (S)-(+)-3-hydroxybutyrate all also elicited responses at the elution time of ethyl acetate (Fig. 2). These responses indicated that ethyl acetate might have been a trace impurity in these stimuli. The responses were quite different in size for the different stimuli, indicating that the contamination differed in concentration. Indeed, other stimuli that we tested did not elicit any response at the elution time of ethyl acetate (see, for example, the response to methyl acetate or to 3-hexanone in Figure 2), indicating that these responses must have been generated by a specific impurity. Other impurities also elicited responses: ethyl butyrate elicited four response peaks in Or42b (Fig. 2), one with retention time corresponding to ethyl acetate, one with retention time corresponding to ethyl propionate or propyl acetate, one unknown, and one corresponding to ethyl butyrate itself.

What was the concentration of the ethyl acetate contamination in the benzaldehyde-d_5_ sample? We recorded a dose-response curve of ethyl acetate calcium responses in Or42b from the purified GC eluate. At very low concentration, no response could be detected. With increasing concentration, the response size increased, and at very high concentration the response formed a tail, with calcium decreasing only slowly (Fig. 3*A*, red traces). Across concentrations, this yielded a sigmoidal dose-response curve, with half-maximal response at a dilution of 10^−5.0^ (Fig. 3*B*). We normalized these responses to the ethyl acetate peak in the response to ethyl butyrate (Fig. 3*A*, gray trace). The responses to the benzaldehyde-d_5_ concentration were weaker (Fig. 3*C*, green traces, corresponding to benzaldehyde-d_5_ dilutions of 10^−2^, light trace, and 10^−1^, dark trace). These responses corresponded to the values for ethyl acetate of 10^−7.4^ and 10^−6.1^, in good approximation of a single decadic dilution step. Thus, we could quantify that a 10^−1^ dilution of benzaldehyde-d_5_ contained 10^−6.1^ ethyl acetate, while a 10^−2^ dilution contained 10^−7.4^ ethyl acetate, on average a 10^−5.2^ contamination. This corresponded to an impurity of 6 ppm, or 0.0006%, which is at the low end of the detection limit of gas chromatography using flame ionization detectors.

Could the heat in the GC cause unexpected artefacts, such as conformational changes in the molecules? To exclude this possibility, and to test whether artificially adding an impurity of ethyl acetate to benzaldehyde-h is sufficient to generate a response as the one that we found for benzaldehyde-d_5_, we generated synthetic mixtures of benzaldehyde-h with the impurity. We recorded the calcium responses in Or42b > GCaMP6m antennae. Responses to benzaldehyde-h were again inhibitory (Fig. 4, trace 0). Adding increasing concentrations of ethyl acetate ranging from 10^−10^-10^−2^ led to a dose-dependent shift from the inhibitory response (Fig. 4, traces 10^−10^-10^−7^) to an increasingly excitatory response (Fig. 4, traces 10^−6^-10^−2^), confirming that adding minute amounts of ethyl acetate was sufficient to mimic the response induced by benzaldehyde-d_5_.

## Discussion

Many olfactory receptors have a broad response profile, with sensitivities ranging over many log-decade concentrations. For example, the *Drosophila* receptor Or22a has a half-maximal response to methyl hexanoate at a dilution of 10^−6.9^, and to isoamyl acetate at a dilution of 10^−4.2^ (Pelz et al., 2006; note that quantitative indications of concentrations depend on experiment specific settings, therefore, absolute values are difficult to compare between experiments; relative values, however, are comparable). Both are substances and concentrations that occur in the environment of the fruit fly, therefore, both are ecologically relevant. This gives an interesting twist to analyzing odorant responses in a natural environment, where most stimuli are mixtures of several chemicals: a response might derive from a major component, from a trace element, or both (Münch et al., 2013).

Here, we give an example where an impurity of 0.0006% (6 ppm) explains the full response of a single receptor cell type. Given that for most substances the highest commercially available purity is 95% or 99%, these results are important for our interpretation of many odorant-response studies, and not limited to investigating the vibrational theory. The headspace of the benzaldehyde-d_5_ batch that we used in our experiments had been analyzed chemically in great detail, resulting in 99.85% purity, with a 0.1% impurity due to an individual contaminant, but no evidence for ethyl acetate (data not shown), since the GC analysis did not reach the 0.0006% sensitivity that the natural *Drosophila* receptor has. Another study used benzaldehyde-d_6_, and the chemical analysis revealed eight contaminants, all of which at a concentration higher than 0.0006% (Drimyli et al., 2016). Under such circumstances, the contribution of ethyl acetate can easily go undetected when testing deuterated benzaldehyde. Furthermore, ethyl acetate is not used in the synthesizing process of benzaldehyde-d_5_ (personal communication from the manufacturers), adding the additional caveat that post-production impurities could be any chemical. We do not claim that any particular study about the effect of deuterated substances can be explained by trace impurities. For example, experiments showing learning transfer between deuterated compounds and nitriles (Franco et al., 2011) are less likely to suffer from an impurity problem. We can only add a note of caution, and substantiate the need for on-the-spot purification. Furthermore, trace compounds, even if they are good ligands when given alone, do not always dominate the response of a receptor in a mixture: a “secondary” ligand given simultaneously in a mixture could be able to obscure the response to the primary ligand due to syntopic interactions (Münch et al., 2013). In such a case, the response to the trace component would be visible when purified (e.g., as done here, with the GC), but it would not contribute significantly to the response when given in a mixture, as contaminant.

Examples of highly sensitive olfactory receptors have been published previously: several moth species have receptors highly sensitive and selective for (-)-germacrene-D, and give responses to stimulation down to 1 ng, and 10-fold less sensitive (10 ng) to the enantiomer. In these recordings, tiny amounts of (-)-germacrene-D among other substances created false positive results in physiologic recordings in moths (Stranden et al., 2003). To ensure purity of the delivered stimulus, it is necessary to record from the olfactory receptor at the exit of a gas chromatographic column (Stranden et al., 2003; Schubert et al., 2014). This technique has been used to identify other highly selective and sensitive receptors (Stensmyr et al., 2012; Dweck et al., 2013; Ebrahim et al., 2015).

Odors are encoded as combinatorial patterns of activated olfactory receptors (Galizia, 2014). Therefore, it is necessary to measure the responses of many receptor neurons to many chemical substances, an approach that has been performed in a series of screening experiments, many of them in *Drosophila* (Hallem and Carlson, 2006; Kreher et al., 2008; Montague et al., 2011; Silbering et al., 2011). These have been collected in a consensus data-base (Münch and Galizia, 2016) that allows for computational analyses of odor coding (Boyle et al., 2013; Saberi and Seyed-allaei, 2016). However, the results here add a note of caution to the reliability of large odor-response screens. Out of the ten substances tested in Figure 2 for Or42b, four (ethyl propionate, ethyl butyrate, propyl acetate, and ethyl(S)-(+)-3-hydroxybutyrate) gave responses not only to the main component, but also to a (small) contamination with ethyl acetate. Importantly, ethyl acetate was not the only trace impurity to elicit responses (see responses to 3-penten-2-one and responses to ethyl butyrate, that had two more effective impurities, one putatively propyl acetate). These minute contaminations create a distortion in large screening studies that is difficult to correct without reassessing all measurements in a GC-coupled mode. In the specific case of Figure 2, for example, we tested the ten best ligands according to the consensus database in DoOR (Münch and Galizia, 2016). The best ligand in our data were ethyl acetate (Fig. 2). In the DoOR database ethyl acetate does not rank first, since not all studies of Or42b reported ethyl acetate as the strongest ligand, and the merging algorithm in DoOR is agnostic about the reliability of each study. Some of the differences, e.g., in the case of ethyl (S)-(+)-3-hydroxybutyrate, may be due to differences in concentrations used across studies (most screening studies do not include full concentration series). However, some “best ligands” in the database may have been overvalued due to the contribution of a contaminant in the chemical sample.

We started this study searching for a receptor that would respond differently to a deuterated substance than to the hydrogenated substance, in the case of a positive result, this would have indicated that that receptor might have been sensitive to a vibration around 550 cm^−1^ or around 2150 cm^−1^. While we found a receptor that responded differently to our two stimuli, we could show that this difference was due not to the deuteration, but rather to a minute impurity of 0.0006%, while the response to deuterated benzaldehyde was identical to the response to hydrogenated benzaldehyde (Fig. 1*E*). By adding the impurity to benzaldehyde-h we obtained the same response as for the contaminated benzaldehyde-d_5_, confirming that the contamination was sufficient to overcome the inhibitory effect of benzaldehyde-h and induce an excitatory response (Fig. 4). We can show that Or42b is not responding to a vibration of 550 or 2150 cm^−1^, and it is unlikely that any of the ORs labeled in an Orco line are responding to that vibration in benzaldehyde-d_5_ either, because such a difference would have been seen in our measurements of the antennal lobe (Fig. 1*C*). These results do not exclude that there might be receptors in *Drosophila* (or other species) that have evolved a mechanism for using molecular vibration to support response selectivity.

## Synthesis

Reviewing Editor: Zoltan Nusser, Institute of Experimental Medicine Decisions are customarily a result of the Reviewing Editor and the peer reviewers coming together and discussing their recommendations until a consensus is reached. When revisions are invited, a fact-based synthesis statement explaining their decision and outlining what is needed to prepare a revision will be listed below. The following reviewer(s) agreed to reveal their identity: Mihaly Kollo, Richard Gerkin.

The paper raises important caveats for experiments studying odor responses at the receptor level. The authors point out rightly that potential artifacts due to low concentration contaminants can interfere with the results of receptor tuning studies. Most studies of neural activation of an odorant rely on the assumption that the composition of the odorant reflects the dominant molecule (i.e. if an odorant is 99.99% pure n-heptanol, then it is assumed that n-heptanol is driving any measured result). This manuscript shows that this assumption is invalid, and that observed data, past, present, and future, can sometimes be entirely explained by molecular components present at only a few parts per million. Consequently, these results indicate the need of re-evaluating published data. The Reviewers and I found that this is an exciting work, which raises a very important issue. There are only few issues the authors should address before the MS can be published.

The study raises an important issue regarding experiments comparing odor responses to deuterated and undeuterated compounds, demonstrating that very low level impurities in commercial high-purity substances have significant effects on olfactory receptor responses. The authors rightly point out that this also needs to be considered during the large-scale screening of receptor tuning. The study employs a well suited experimental approach for the detailed, quantitative analysis of receptor affinity, and the results are generally convincing. On the other hand, the manuscript provides no statistical analysis apart from the s.e.m. on the plots, and the sample sizes are small.

(1) Despite the claim that the study pinpointed the reason for the difference in deuterated and undeuterated benzaldehyde responses of Or42b, there was no direct experiment showing that a mixture benzaldehyde-h and the correct amount of ethyl acetate reproduces the responses to benzaldehyde-d5. This would help to provide a link between direct odor application and GC-coupled experiments, which operate at very different timescales.

(2) The extremely low concentration of identified impurities raises the question whether thermal degradation of the tested compounds could contribute to the composition of the eluate. This question is not addressed in the manuscript, and only a single set of parameters (flow, temperature) was tested during the GC coupled recordings. This issue should be addressed in the MS.

Minor points:

1) The color scale (red-yellow-white) of the calcium response in Figure 1C overlaps with the background anatomy image (black-white).

2) Page 12: “can be able”

3) Is the temperature (200 degrees) at the GC output of any concern, i.e. could any reaction occur (even under inert gas, maybe a conformational change) that could change the identity of the substance entering the PID versus the nominally identical substance reaching the insect antenna?

4) Presumably this work was initially motivated by the idea of testing the vibrational hypothesis, whereas the impurity issue ultimately led you to the present the work from a different angle. Early in the paper you seem to be pushing more ambitiously the idea that the impurity issue undermines the vibrational hypothesis, whereas later you pull back somewhat and acknowledge that it neither confirms nor refutes it, and also that some other evidence for the vibrational hypothesis may be difficult to explain as an artifact of impurities. I don't know what the right answer is, but I would encourage you to take a moment and assess the overall impact of your result on the plausibility of the vibrational hypothesis, and try to make the tone of the manuscript, when speaking of that hypothesis, a bit more consistent throughout with your take on that. Also, in fairness, there are probably other results from olfaction that are unrelated to the vibrational hypothesis that may be undermined or re-evaluated in light of your result. You should discuss these as well, since the fact that you initially aimed to test the vibrational hypothesis shouldn't lock you in to discussing the implications only for that hypothesis. For example, what about all other receptor-ligand binding studies in the olfactory system, including in mammals? How many of these need to be reevaluated? What about psychophysical studies with nominally pure odorants?
